# Dynamic monitoring of platelet activation and its role in post-dissection inflammation in a canine model of acute type A aortic dissection

**DOI:** 10.1186/s13019-016-0472-5

**Published:** 2016-05-26

**Authors:** Chaoyi Qin, Hongwei Zhang, Jun Gu, Zhenghua Xiao, Qin Yang, Wei Meng

**Affiliations:** Department of Cardiovascular Surgery, West China Hospital, Sichuan University, Lane outside the southern No. 37, Cheng du, Sichuan, People’s Republic of China; Department of Radiology, West China Hospital, Sichuan University, Lane outside the southern No. 37, Cheng du, Sichuan, People’s Republic of China

**Keywords:** Platelet activation, Acute type A aortic dissection, Inflammation

## Abstract

**Background:**

To confirm the activation of platelets (PLT) and explore the role of activated PLT in post-dissection inflammation.

**Method:**

An acute type A aortic dissection (AAD) canine model was established. Mean platelet volume/platelet count (MPV/PTC), platelet size distribution width (PDW), and inflammatory cytokines (tumor necrosis factor-α [TNF-α] and interleukin-6 [IL-6]) were measured between anesthetization and thoracotomy (T1), at the end of the operation (T2), and at 2 h (T3), 4 h (T4), and 6 h (T5) after the operation. Bivariate analysis was used to determine the correlations between the peak MPV/PTC, PDW, and inflammatory cytokines at T4.

**Result:**

An AAD canine model was successfully established. Both MPV/PTC and PDW values were significantly higher at T3-T5 than at T1 (*P* < 0.05). Both were also significantly higher at T3-T5 in the dissection group than in the sham operation (SO) group (*P* < 0.05). Inflammatory cytokine levels were remarkably higher at T3-T5 than at T1, and were higher at T3-T5 in both the dissection and the SO group (*P* < 0.05). Bivariate analysis demonstrated positive correlations between MPV/PTC and both TNF-α (*r* = 0.826, *P* = 0.011) and IL-6 (*r* = 0.806, *P* = 0.016).

**Conclusion:**

Activated PLT were identified after AAD, and played a critical role in the initiation of post-dissection inflammation.

## Background

Aortic dissection is a life-threatening medical emergency associated with high mortality and morbidity. The 24-h mortality rate is greater than 35 %, and more than half of patients die within 48 h [1]. Previous studies revealed inflammatory changes in the aortic wall and plasma during the course of aortic dissection [2]. However, an effective therapeutic strategy targeting inflammation-associated aortic dissection has not been fully elucidated. Platelet(PLT) activation plays an important role in inflammation and can influence both innate and adaptive immunity [3]. We chose mean platelet volume/platelet count (MPV/PTC) and platelet size distribution width (PDW) as markers for PLT activation. Due to the variable times between onset of symptoms and admission, we needed a standard time-control animal model to simulate the pathological and physiological changes of acute type A aortic dissection(AAD) in patients. In this study, we used an AAD canine model to confirm whether PLT were activated, and to explore their role in inflammation.

## Methods

### Animals and surgical preparation

**Fig. 1:**
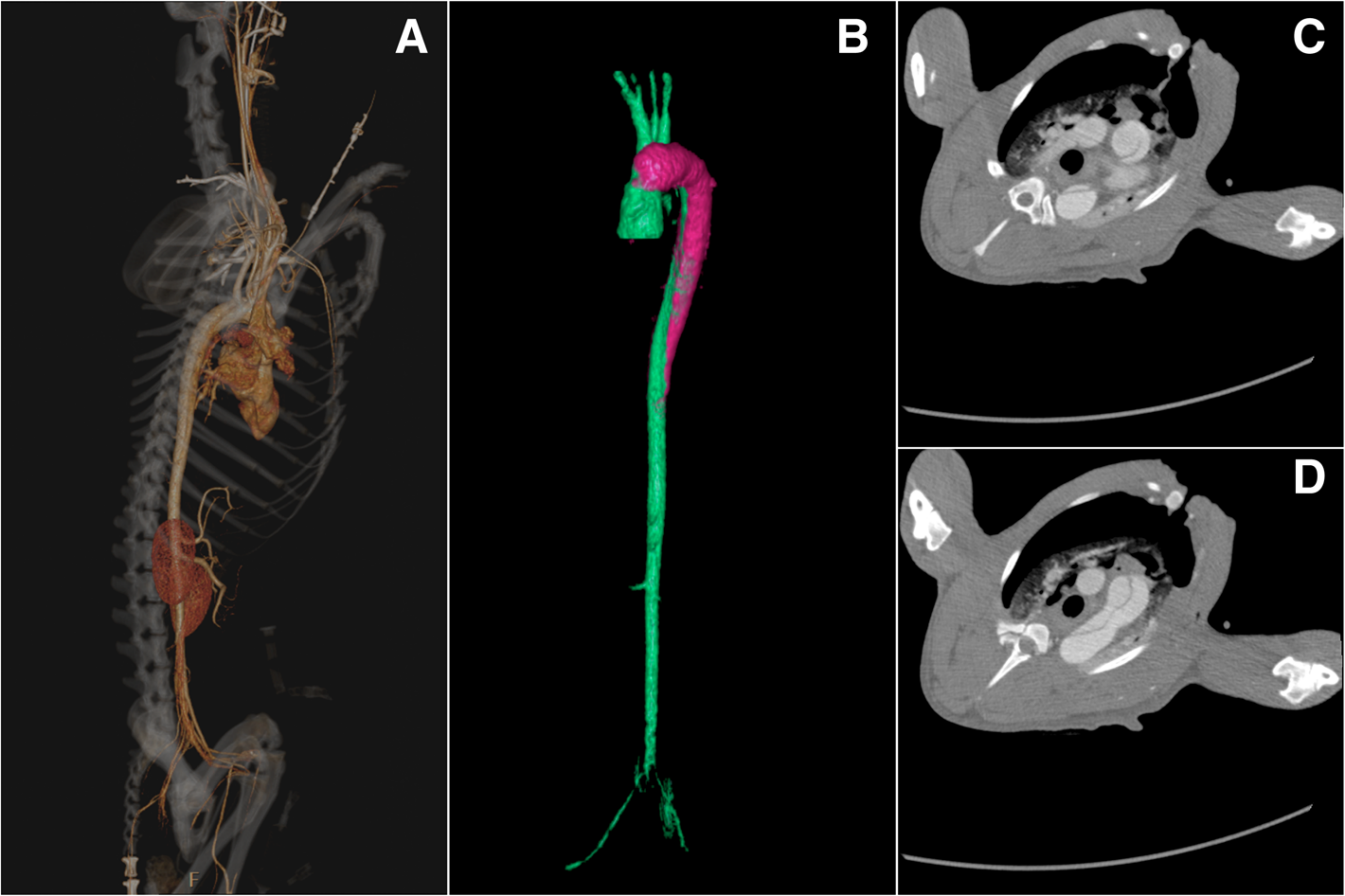
Three-dimensional reconstruction images (**a**, **b**) and computed tomography in cross-sectional views (**c**, **d**) demonstrated in the AAD canine model: the tear entry site located in the ascending aorta; the false lumen was obvious and compressed the true lumen

Sixteen male, healthy adult Beagles with body weights of 9–11 kg were housed separately at temperatures of 23–25 °C. They were fed in the West China Clinical Medical College of Sichuan University/The Experimental Animal Center of West China Hospital, following the regulations for experimental animal welfare. Canines were randomly divided into sham operation(SO) and dissection groups, and were subjected to median thoracotomy alone, or sternotomy with ascending aorta clamping and an aortic dissection surgical procedure, respectively,as previously reported [2]. Briefly, after routine sterile preparation and draping, a median sternotomy was performed to expose the ascending aorta. After the pericardium was elevated, the aortic wall was clamped at about 2 cm distal to the origin of the ascending aorta. A small round blade was used to cut across the adventitia and part of the media. The aortic clamp was loosened after the exposure of the medial space, followed by gentle and blunt extension of the medial space about 1 cm distally with the tip of a mosquito clamp, creating the initial false lumen. Then, the aortic wall was clamped again, and the remaining media and intima were excised, creating a communication between the false lumen and the aortic cavity. The torn intima was fixed with 6–0 Prolene to the opposite aortic wall, which helped the intima resist the force of blood flow and kept the false lumen open. After closure of the aortic wall to allow for continuous blood infusion into the false lumen, the wall clamp was carefully loosened. Finally, the chest wall was closed to finish the procedure, and the aortic dissection canine model was established. Animals in the SO group only underwent a median sternotomy, with no procedures on the aorta. Three-dimensional reconstruction images (Fig. 1a, b) and computed tomography in cross-sectional views (Fig. 1c, d) were used to demonstrate the AAD canine model.

### Blood sample collection and plasma isolation

Blood samples were collected in anticoagulation tubes between anesthetization and thoracotomy (T1), at the end of the operation (T2), and at 2 h (T3), 4 h (T4), and 6 h (T5) after the operation. Whole blood was centrifuged at 1000 rpm/min for 15 min at 4 °C, and supernatant plasma was collected. All plasma samples were carefully stored at −80 °C.

### Parameter measurements

PTC, MPV, and PDW were measured by using a multi-function automated blood cell analyzer (XT-4000i, Sysmex, Japan). Levels of tumor necrosis factor-α (TNF-α), interleukin-6 (IL-6), and IL-10 in the plasma were measured by using enzyme-linked immunosorbent assay (ELISA) techniques (R&D Systems, Minneapolis, MN, USA). All procedures followed standard protocols (included in the ELISA kits). Spectrophotometry was used to detect the intensity of transmitted light. Data were expressed as ng/mL.

### Statistical analysis

All descriptive data were shown as mean ± standard error of the mean (SEM). Multiple comparisons were analyzed by one-way analysis of variance (ANOVA), followed by Bonferroni’s test. The Pearson correlation coefficient test was performed. *P* < 0.05 was considered to be statistically significant.

## Results

### Baseline information

Animal characteristics are presented in Table 1. None of the background conditions (weights, heart rate, systolic blood pressure, diastolic blood pressure, length of aorta) were significantly different (*P* > 0.05) between the two groups. White and red blood cell count, neutrophils, PTC, MPV, PDW, glucose, and total cholesterol before surgery, and dissection length, surgical time, and aortic cross-clamping time were not significantly different (*P* > 0.05).

**Table 1:** Basic information

	Control group	Dissection group
Weight (kg)	9.3 ± 0.9	9.3 ± 0.7
Heart rate (bpm)	146 ± 12	151 ± 16
Systolic blood pressure (mmHg)	167d p	172 ± 28
Diastolic blood pressure (mmHg)	97 ± 10	95 ± 18
Aortic length (cm)	35.1 ± 2.5	34.8 ± 2.1
Laboratory data		
White blood cell (10^9^/L)	13.2 ± 2.1	13.6 ± 1.9
Red blood cell (10^12^/L)	5.9 ± 0.9	5.8 ± 1.0
Neut. (10^9^/L)	0.5 ± 0.3	0.5 ± 0.2
PTC (10^9^/L)	470.1 ± 98.1	469 ± 93.6
MPV (fL)	11.3 ± 3.1	11.7 ± 3.3
PDW (%)	16.4 ± 2.9	16.1 ± 2.6
Glucose (mmol/L)	4.8 ± 0.6	4.5 ± 0.7
Total cholesterol (mmol/L)	3.9 ± 0.8	3.7 ± 0.9
Surgical data		
Dissection length (cm)	-	22.1 ± 1.5
Surgical time (h)	1.3 ± 0.5	1.8 ± 0.4
Aortic cross-clamping time (h)	1.0 ± 0.2	1.0 ± 0.3

All data are presented as mean ± standard deviation (SD). Statistical analysis shows no significant difference between the two groups (*P* > 0.05)

*PTC* platelet count, *MPV* mean platelet volume, *PDW* platelet size distribution width, *Neut.* neutrophil

### Dynamic changes in PLT indices

**Fig. 2:**
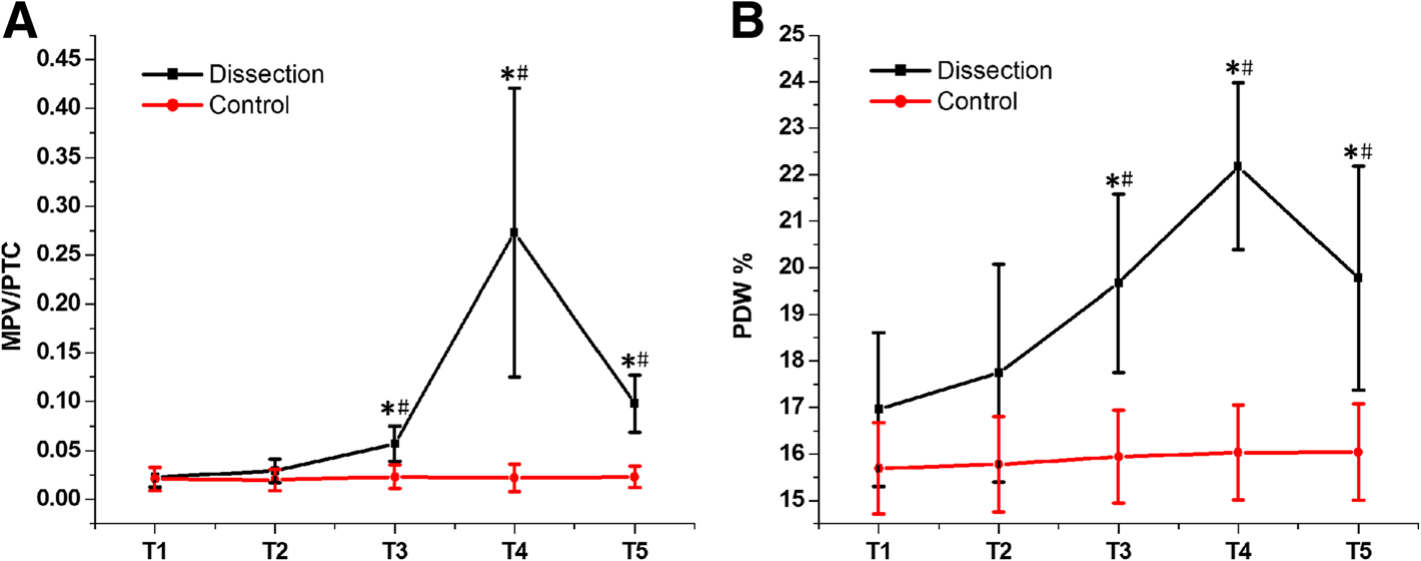
Dynamic changes in MPV/PTC and PDW. **a** MPV/PTC value was significantly greater at T3-T5 than at T1 (*P* < 0.05). It was also remarkably higher at T3-T5 in the dissection group than in the SO group (*P* < 0.05). **b** PDW value was significantly higher at T3-T5 than at T1 (*P* < 0.05). It was also remarkably higher at T3-T5 in the dissection group than in the SO group (*P* < 0.05)

PTC, MPV, and PDW values were measured at all time points to dynamically monitor the activation of PLT. As shown in Fig. 2, MPV/PTC and PDW values were significantly higher at T3-T5 than at T1(*P* < 0.05). Meanwhile, MPV/PTC and PDW values were remarkably greater in the dissection group than in the SO group at T3-T5 (*P* < 0.05). These data indicated that PLT were activated at 2 h after the operation, and reached a peak at 4 h after the operation.

### Dynamic changes in plasma inflammatory cytokines

**Fig. 3:**
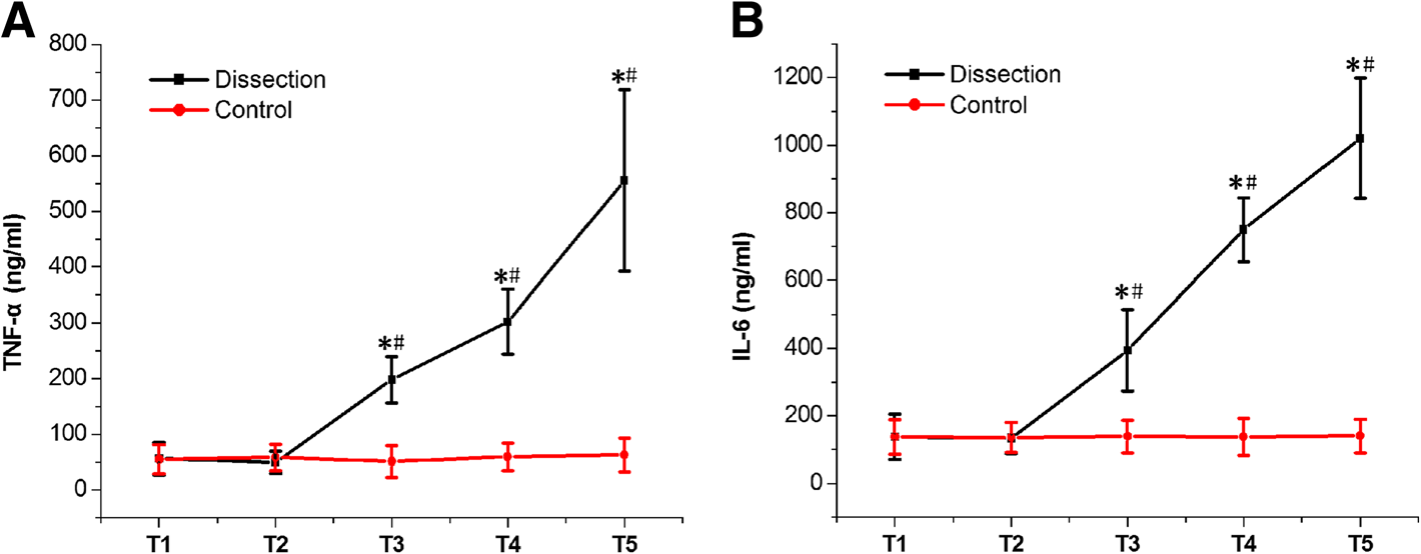
Dynamic changes in TNF-α and IL-6. **a** TNF-α level was significantly higher at T3-T5 than at T1, and was also remarkably higher at T3-T5 in the dissection group than in the SO group (*P* < 0.05). **b** IL-6 level was significantly higher at T3-T5 than at T1 (*P* < 0.05), and was also remarkably higher at T3-T5 in the dissection group than in the SO group (*P* < 0.05)

TNF-α and IL-6 were chosen to represent systemic inflammatory status. Interestingly, after the T2 time point, both TNF-α and IL-6 in the dissection group significantly climbed, and reached highest levels at T5 (*P* < 0.05, Fig. 3a). Similar to the changes in PLT indices, both TNF-α and IL-6 at T3-T5 in the dissection group were significantly higher than in the SO group (*P* < 0.05, Fig. 3a). Given these patterns, which were similar to the PLT indices, the data strongly suggested that systemic inflammation was present2 h after the operation; there was a close relationship between the activation of PLT and the initiation of systemic inflammation.

### Correlations between PLT indices and inflammatory cytokines

**Fig. 4:**
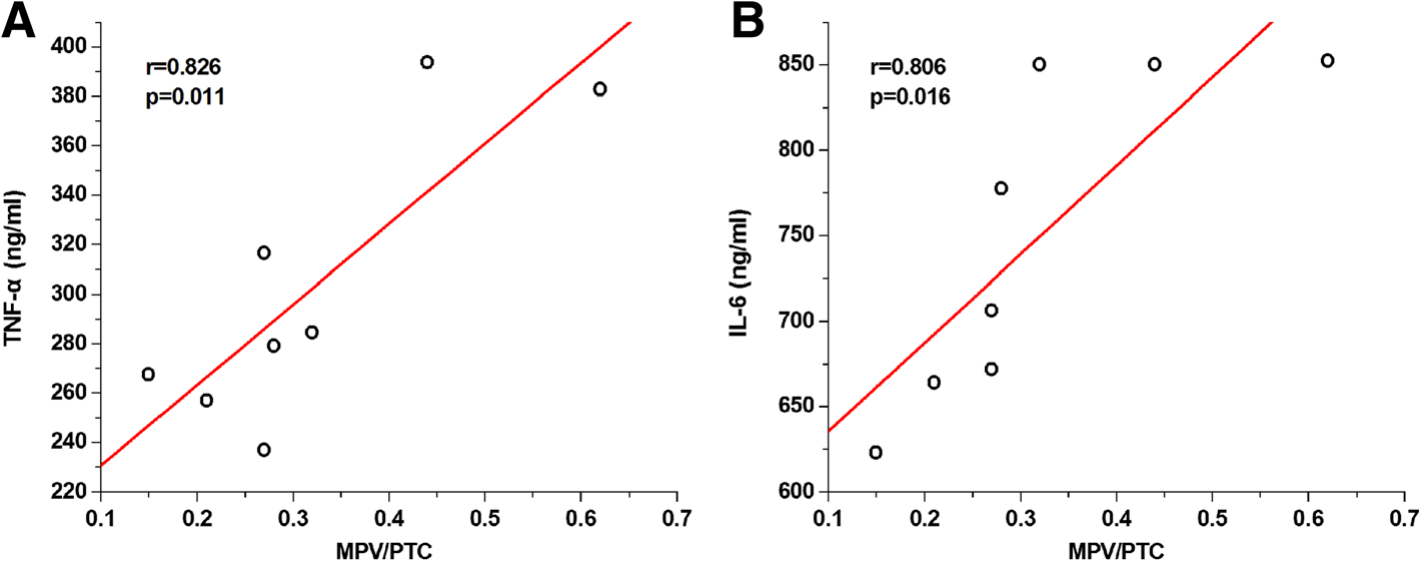
Bivariate analysis comparing peak MPV/PTC and inflammatory cytokines at T4. **a** Bivariate analysis demonstrated that peak MPV/PTC was positively correlated with TNF-α (*r* = 0.826, *P* = 0.011). **b** Bivariate analysis demonstrated that peak MPV/PTC was positively correlated with IL-6 (*r* = 0.806, *P* = 0.016)

To define the relationship between the activation of PLT and the initiation of systemic inflammation, bivariate analysis was used. We studied the correlations between peak MPV/PTC and PDW levels, and inflammatory cytokines at T4. Unsurprisingly, positive correlations between peak MPV/PTC and both TNF-α (*r* = 0.826, *P* = 0.011, Fig. 4a) and IL-6 (*r* = 0.806, *P* = 0.016, Fig. 4b) were confirmed and are shown in Fig. 4. However, although there were still rough but positive correlations between peak PDW and both TNF-α (*r* = 0.623, *P* = 0.082) and IL-6 (*r* = 0.603, *P* = 0.069), there were no statistically significant differencesto explain these data.

## Discussion

Our study established an AAD canine model, which followed our previous procedures. With this new animal model, we first dynamically monitored the level of PLT activation and the systemic inflammatory level. Intriguingly, the same start points for PLT activation and systemic inflammation were identified. Furthermore, bivariate analysis demonstrated that peak MPV/PTC ratios were positively correlated with inflammatory cytokine sat T4 in the dissection group.

Many studies have reported reduced PTC and increased MPV in unruptured abdominal aortic aneurysms [4]. There is an increased MPV/PTC in patients with aortic dissection compared to normal controls [5]. This suggests that activation of PLT is present in patients with aortic aneurysms and dissection. Our data first confirmed and reported an increased MPV/PTC and PDW in AAD canine models, which characterized the presence of activation of PLT. AAD is also characterized by systemic inflammatory response syndrome (SIRS), which may worsen the AAD and affect the prognosis [6, 7]. Levels of TNF-α, IL-6, and IL-10 reportedly represent the severity of inflammation after AAD [8]. Although many measures have been taken to diminish these systemic inflammatory responses, there is still no effective means of doing so [9]. In the present study, we observed systemic inflammation after the operation. Significant increases in TNF-α and IL-6 were seen 2 h after the operation, and climbed still further. Unexpectedly, T3 was identified as the same starting point for both inflammatory cytokines and activation of PLT, which directed us to further study the relationship between the activation of PLT and systemic inflammation.

It is well known that the activation of PLT plays an important role in initiation and progression of inflammation [10]. Activated PLT release many pro-inflammatory cytokines, such as IL-1, IL-6, platelet activating factor, and P-selectin [11, 12]. Additionally, IL-1 can induce the expression of adhesion molecules, promoting the adhesion of monocytes and endothelial cells, and the release of TNF-α and IL-6 [13]. Given the pro-inflammatory features of PLT, it was even suggested that PLT on admission can be a predictor of mortality [14, 15]. All these factors suggested that activated PLT might have a critical role in initiation and progression of post-dissection inflammatory responses. In our study, we identified the same starting point for activation of both PLT and inflammatory cytokines. To further understand this relationship, bivariate analysis was performed. Positive and significant correlations between inflammatory cytokines and activation of PLT were demonstrated. This strongly suggested that the activation of PLT can cause severe inflammatory responses in AAD. Additionally, our study provided new insights into a mechanism for prevention of inflammatory responses in AAD.

## Conclusion

Our study did not examine the entire range of inflammatory cytokines. It is well known that many other factors may worsen post-dissection inflammation. In the present study, we focused on the activation of PLT, and postulated its greater responsibility in the initiation, rather than the progression, of post-dissection inflammatory responses. Our study suggested a new approach to manage post-dissection inflammation, and thereby to improve the prognosis of AAD.
